# Bird-plant frugivory networks in two secondary *Araucaria* forests in southern Brazil

**DOI:** 10.1371/journal.pone.0357566

**Published:** 2026-09-30

**Authors:** Francisco A. Vásquez-Arévalo, Pamela E. Malmoria, Carla S. Fontana, Pedro Maria Abreu Ferreira

**Affiliations:** 1 Dirección de Investigación en Diversidad Biológica Terrestre Amazónica (DBIO), Instituto de Investigaciones de la Amazonía Peruana (IIAP), Iquitos, Loreto, Perú; 2 Departamento de Ecología, Genética y Evolución & IEGEBA-CONICET, Facultad de Ciencias Exactas y Naturales, Universidad de Buenos Aires, Pabellón II Ciudad Universitaria, Buenos Aires, Argentina; 3 Programa de Pós-graduação em Biodiversidade Animal, Centro de Ciências Naturais e Exatas, Universidade Federal de Santa Maria (UFSM), Santa Maria, Rio Grande do Sul, Brazil; 4 Departamento de Ecologia, Universidade Federal do Rio Grande do Sul (UFRGS), Porto Alegre, Rio Grande do Sul, Brazil; 5 Laboratório de Ecologia de Interações, Programa de Pós-Graduação em Ecologia e Evolução da Biodiversidade, Pontifícia Universidade Católica do Rio Grande do Sul (PUCRS), Porto Alegre, Rio Grande do Sul, Brazil; Universidade Federal de Minas Gerais, BRAZIL

## Abstract

The properties of mutualistic networks may vary depending on the habitat structure in which partners interact. In this research, we evaluated two plant-bird frugivorous networks from the South Brazilian Atlantic Forest (*Araucaria* Forest) and tested how different past disturbance history and present successional stage can influence the structure of these networks. We sampled six secondary forest sites within a protected area in Rio Grande do Sul, Brazil—three in early succession following clear-cutting (CC), and three in late succession following selective logging (SL), all with similar disturbance timing in the past. Bird feces were collected from individuals captured with mist nets to identify plant–bird interaction events. We constructed bipartite networks from quantitative matrices and compared network- and species-level descriptors across habitats. We identified 19 bird species interacting with 11 plant species in the CC network, and 13 bird species with 10 plant species in the SL network. *Myrsine lorentziana*, an important pioneer species for the local forest ecosystems, *was* a key species in both networks, concentrating the highest number of bird interactions. Both networks were more specialized and modular than expected by chance but did not exhibit significant nestedness. Specialization was generally low, with no significant difference between networks. Modularity was higher in the CC network. On average, plants were more specialized than birds in both networks. Bird abundance did not correlate with species-level specialization, but it did correlate with the number of interactions in the SL network. The same two species, *Myrsine lorentziana* (plant) and *Turdus albicollis* (bird), were central in both networks. Additionally, migratory birds were part of the core group of interacting species. Our results showed that plant-bird mutualistic networks of frugivory were structured according to forest type, differing in modularity, composition, and species centrality. Furthermore, the persistence of *Myrsine lorentziana* and Turdidae as central nodes of interaction throughout successional stages suggests their key role in stability and connectivity in the disturbed remnants of the *Araucaria* Forest.

## Introduction

Mutualistic interactions between plants and animals, including frugivory, contribute to the maintenance of biodiversity by facilitating seed dispersal for plants and providing food resources for animals. [1]. In forest ecosystems, the structure of these interactions can vary significantly depending on the disturbance history and the successional stage of the habitat [2,3]. Specifically, forest regeneration, influenced by past disturbances such as selective logging and clear-cutting, can alter the composition of plant and bird species, directly affecting frugivory patterns and seed dispersal dynamics [2,4–6].

Deforestation and other disturbances alter habitat structure, which in turn affects species diversity and interspecific interactions [2,7]. For instance, in the Atlantic Forest, a mosaic of secondary forests with different intervention histories exhibits significant changes in plant [8,9] and bird communities [3,10], as well as species diversity across habitats [2,3,9,11].

While there is a growing body of research on plant-animal interaction networks, the majority of studies have focused on pollination networks, which are well represented in the literature (e.g., [12,13], compared to studies of frugivory networks. The current evidence, albeit limited, highlights the importance of examining these networks to understand how ecological processes function under different disturbance histories and successional stages [2]. Notwithstanding, the application of network theory to these questions remains limited, especially considering tropical and subtropical ecosystems from southern South America.

Using a network approach to study interactions such as frugivory provides effective tools for understanding how ecological processes respond to disturbance histories and successional changes. This approach allows the assessment of community-level emergent properties, such as nestedness, specialization, and modularity, which may vary depending on the forest's successional stage. Along a disturbance gradient of the Atlantic Forest in southeastern Brazil, variations in frugivory interaction patterns have been observed; for example, networks show greater nestedness in smaller disturbance areas, while their specialization is invariant along the fragmentation gradient [2]. Other studies on herbivory networks have reported that modularity tends to be low in grassland areas compared to early and later regeneration stages. However, modularity is not entirely different between early regeneration stages and primary forest [14,15].

We present a study that describes and compares the structure of two frugivory networks between birds and plants in two secondary Araucaria forests in southern Brazil. While one site underwent a regeneration process following clear-cutting for livestock activities, the second one is regenerating after selective logging. Both networks were compared using the following descriptors: network size, nestedness, specialization, modularity, and species centrality. Additionally, we evaluated the contribution of local bird abundance to their degree of interaction and specialization within each network. Based on the premise that the successional history of forest sites determines habitat structure, i.e., plant community structure [8,16–18], which in turn influences the structure of bird communities [3,10,19], we hypothesize that the disturbance histories of two secondary Araucaria forests in southern Brazil influence the structure of bird-plant frugivory networks. For this hypothesis, we have the following predictions: (1) the forest showing more plant species will comprise a network with more birds and observed interactions [9,10], (2) the network assembled in the site with more plant and bird species will be more nested in comparison with its species-poorer counterpart [7], (3) the network with more generalist species will have a lower degree of specialization at the network level [20], (4) networks with higher number of species and interactions will be more modular [21–23], and (5) the central and peripheral species within the network will change depending on the forest type [24–26]. Additionally, based on the premise that mutualistic interactions can result from neutral and/or niche processes [22,27,28], we hypothesize that local bird abundance will be a good predictor of their degree and specialization. For this hypothesis, we make the following predictions: if interactions are the product of stochastic and density-dependent processes, species with higher relative abundance will have a higher degree of interaction and lower specialization. Conversely, if interactions are more related to niche processes (e.g., trait matching), species degree and specialization will not be predicted by abundance.

## Methods

### Study area

The present study was conducted from October 18, 2019, to March 20, 2020, at the Reserva Particular do Patrimônio Natural Pró-Mata (RPPN Pró-Mata) located at coordinates 29°26’17"S - 29°34’42"S and 50°08’14"W – 50°14’18"W [29], in the municipality of São Francisco de Paula, Rio Grande do Sul, Brazil (Fig 1). This period includes the reproductive season and arrival of migratory species in southern Brazil [30–33]; it also includes the season of mass fruit production for many plant species in the área [34–36].

**Fig 1:**
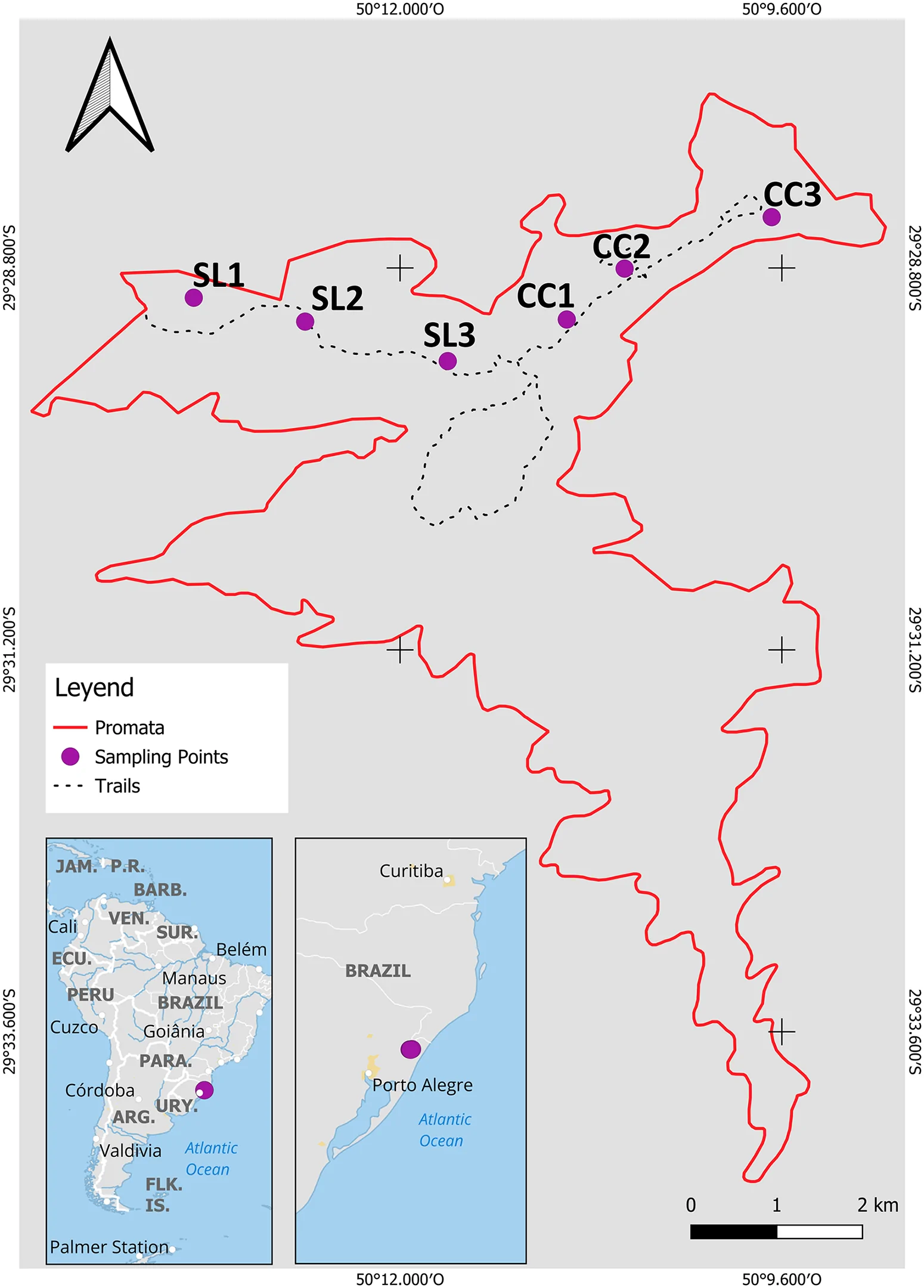
Map showing the location of sampling units in the RPPN Pró-Mata, municipality of São Francisco de Paula, Rio Grande do Sul, Brazil. Purple dots indicate sampling units in early successional forest after clear cutting (CC1, CC2, CC3) or in the late successional forest after selective logging (SL1, SL2, SL3). Base map from Natural Earth (public domain).

Pró-Mata is situated within the geomorphological unit called Planalto das Araucárias, which is part of the Serra Geral rock formation [37]. This area features a fluctuating and rugged relief, with steep slopes and an altitudinal variation from 200 to 900 meters above sea level [29]. The climate is humid with an average annual temperature of 14.5°C, dropping to 0°C in winter, and precipitation ranging from 1750 to 2500 mm annually [17,38,39].

Pró-Mata covers a total area of approximately 3,100 ha [25] and includes a mosaic of vegetation formations characteristic of the northeastern region of Rio Grande do Sul, which includes the Mixed Ombrophilous Forest (*Araucaria* Forest), Dense Ombrophilous Forest (Brazilian Atlantic Rainforest), and Highland Grasslands [40–42]. The predominant forest cover in the area corresponds to the Mixed Ombrophilous Forest, also known as the Araucaria Forest, which is currently recovering from partial or total removal of vegetation cover due to livestock activities or selective logging [41,43] (Fig 1). For our study, we selected 03 sampling units in areas undergoing recovery following total vegetation removal and 03 in areas where there had been partial removal of vegetation. For the selection of sampling areas, we used the floristic descriptions of Pró-Mata provided by Vicente [44], Vicente-Silva et al. [9], and Mello [17]. The selected areas have the following characteristics:

**Secondary *Araucaria* forest in early succession following clear-cutting (CC):** These areas are characterized by the prevalence of shrubs, herbs, and small to medium-sized pioneer tree species. Their physiognomy results from natural regeneration after the complete removal of vegetation cover for agricultural and livestock activities ca. 32 years ago. This type of vegetation is locally known as ‘capoeira’; here, canopy height varies, with few large trees and many juvenile *Araucaria angustifolia* (Araucariaceae) present. Pioneer species such as: *Tibouchina sellowiana* (Melastomataceae)*, Drimys angustifolia* (Winteraceae)*, Myrsine lorentziana* (Primulaceae)*, Sapium glandulosum* (Euphorbiaceae) and *Daphnopsis fasciculata* (Thymelaeaceae) [9,17,44].

**Secondary *Araucaria* forest in late succession after selective logging (SL):** In these areas, the impacts of forest activities varied, with selective logging of large trees being the main practice instead of complete deforestation. This type of management has resulted in a successional and structural mosaic in the region. The forest in these areas features an almost uniform canopy with an average height of 15 m, a higher density of large trees, and numerous adult specimens of *Araucaria angustifolia*. In this habitat, species such as: *Myrcia retorta* (Myrtaceae), *Myrceugenia myrcioides* (Myrtaceae)*, Drimys angustifolia* (Winteraceae)*, Ilex microdonta* (Aquifoliaceae)*, A. angustifolia* (Araucariaceae)*, Myrceugenia euosma* (Myrtaceae), among others [9,17,44].

### Interaction records

#### Capturing birds and collecting seeds.

To detect frugivory interactions, we collected seeds from the feces of birds captured at nine sampling units; for this purpose, we used 10 mist nets measuring 12 x 3 m between 05:30 and 18:30 hrs with a 20–30 min inspection period. The birds were captured, identified, and ringed for subsequent release. Bird identification was based on morphological descriptions from the Birds of Brazil and South America guides [45]; likewise, bird nomenclature followed the Comitê Brasileiro de Registros Ornitológicos [46].

We monitored events of bird defecation to collect the feces in paper envelopes; the envelopes were coded with the bir*d’*s ringing code, capture date, bird species, and sampling unit name. The feces were dried in an oven at 37°C for 20 min to facilitate item separation and seed recognition. For seed identification, we used the guides *Sementes de Caratinga* [47] and *Frutos e Sementes de Myrtaceae* [48]. Additionally, we collected seeds from fruiting plants to create a seed bank for comparisons with seeds in feces. Fruiting plants were collected and pressed for identification using the following literature: *A flora de Cambará* [49], *Árvores do Sul* [50] and *Flora arbórea e arborescente do Rio Grande do Sul* [51]. Seeds that could not be identified were grouped into morphospecies.

The research was authorized by the Ministério do Meio Ambiente de Brazil (MMA), Instituto Chico Mendes de Conservação da Biodiversidade (ICMBio), Sistema de Autorização e Informação em Biodiversidade (SISBIO) (Authorization Number: 71472−1, Authentication code: 0714720120191009).

#### Interaction events.

First, we quantified the total number of seeds identified in each fecal sample. To standardize interaction strength across plant species, we defined one interaction event as equivalent to the average number of seeds per fruit, based on published functional trait data for plants from the Pró-Mata region [9]. Thus, when multiple seeds of the same plant species were detected in a sample, the number of interaction events was estimated by dividing the total seed count by the species-specific average number of seeds per fruit. In cases where seeds could only be identified at the morphospecies level and species-level fruit trait data were unavailable, we adopted a conservative approach and defined one interaction event as the average number of seeds recorded per bird individual.

### Data analyses

All analyses were developed using the R programming environment and R-studio integrated development environment [52,53]*.*

#### Diversity, number of interactions and species composition.

First, we created two bipartite networks: clear-cutting network (CC) and selective logging (SL) network, based on quantitative matrices using the number of interaction events [54].

Subsequently, we counted bird and plant richness as the number of species participating per network; likewise, we tallied the number of interaction events. Additionally, we describe the birds’ diet using the foundational database of Wilman et al. [55].

Finally, we conducted a rarefaction analysis using the order q = 0 to estimate species richness for both birds (using the number of captures) and plants (using the number of captures), as well as interactions (using the number of events). The rarefaction analysis displayed a 95% confidence interval for all estimates [56,57]. For this analysis, we utilized the ‘iNEXT’ package [56].

#### Network level metrics.

For network-level comparisons, we utilized the ‘networklevel’ function from the ‘bipartite’ package [58,59] to calculate key network indices: NODF, which ranges from 0 (minimum nestedness) to 100 (maximum nestedness) [59,60], network-level specialization (*H2’*) ranging from 0 (minimum specialization) to 1 (maximum specialization) [59,61], and modularity degree (Q), ranging from 0 (minimum modularity) to 1 (maximum modularity) [58,62].

We created null models using the r2d randomization method [59] by generating 1,000 random networks to test if the observed values of nestedness*,* NODF, *H2’,* and Q were significantly higher than values generated at random [24]. In addition, to evaluate the possible effect of the null model on the estimation of nestedness significance, we ran the same analysis for each metric using four different algorithms (“swap.web,” “vaznull,” “shuffle.web,” and “mgen”) and four nestedness indexes (“nestedness”, “NODF”, “weighted nestedness”, and “weighted NODF”; see S3 and S4 Appendix to see the results). We compared the mean values of *H2’* between both networks based on values obtained from each sampling unit; the comparison was performed using the non-parametric Mann-Whitney test [63]. We used the Z-score values of Q obtained from null models for comparison between networks, to control for differences in network size [59]. The absolute values obtained for each index reflect generalized interaction patterns, while the comparison with the null models assesses whether these patterns differed significantly from random expectations (i.e., whether the observed index significantly departed from the index calculated for all randomly generated networks that comprised the null models).

#### Species-level metrics.

We used the ‘specieslevel’ function from the ‘bipartite’ package [58,59] to obtain the normalized interaction degree (sum of links of a species divided by the number of species at the other level) [58,59] and specialization (*d’*) of each species [58,59,61]. Subsequently, we tested whether the local abundance of birds was a descriptor of their interaction degree and specialization. For this, we performed a Generalized Linear Model (GLM) using the total number of individuals captured throughout the sampling season (i.e., abundance estimation was not limited to sampling events in which interactions with plants were observed) as the explanatory variable (x) and the values of normalized degree and specialization (*d’*) as response variables (y).

Finally, we determined which species were central and which were peripheral in the network by calculating the *Gc* index [25], which classifies species according to their relative connectivity within the network,


$$\begin{array}{c} Gc=\;\frac{{k\;}_{i}-\;k_{mean}}{\sigma k} \end{array}$$

where k_i_ represents the mean interaction frequency of species i, calculated as the average number of plant–bird interaction records per species across the entire bipartite matrix. This metric reflects the effective degree of each species, taking into account repeated interactions rather than simple presence–absence.. k_mean_ corresponds to the mean interaction frequency across all species in the network, and $\sigma_{k}$is the standard deviation of interaction frequencies among species, and σk is the standard deviation of interaction frequencies among species.. Species with Gc > 0 exhibit higher-than-average interaction frequencies and were classified as central, whereas species with Gc ≤ 0 were considered peripheral.

## Results

### Network components by forest type

#### Bird diversity.

Throughout the sampling season, we captured 729 individuals (4200 h/net), corresponding to 74 species and 20 families (S1 Appendix). In the selective logging sites (SL) (2040 h/net) we captured 480 individuals, distributed across 67 species and 17 families, whereas in the clear-cutting sites (CC) (2160 h/net), we captured 249 individuals, distributed across 39 species and 15 families (S1 Appendix).

Out of this total of captures, 17.9% (126 captures) showed interactions with plants. Thus, in the SL sites we detected 19 species with 95 individuals and 7 families, and in the CC sites 13 species participated with 30 individuals and 8 families (S1 Appendix).

#### Plant diversity.

A total of 13 plant species (737 seeds), distributed across 12 families and 10 orders, were consumed. From this set of species, we were unable to identify two types of seeds to the family level (Morph 7 and 13). In the removal forest, we identified 11 plant species (555 seeds) (S2 Appendix), and in the tree felling, we identified 10 species (182 seeds) (S2 Appendix). Additionally, we counted a total of 72 unique links (qualitative count of the specific consumption of a plant species by a bird species): 45 unique links in the SL sites and 27 in the CC sites.

#### Rarefaction of bird, plant and interaction diversity.

Observed bird species richness considering total captures was higher in the SL network in comparison with the CC network, considering both interpolated and extrapolated values (Fig 2a). Considering only captures with plant-bird interaction events, there was no difference between disturbance histories (i.e., confidence intervals overlap) for bird richness (Fig 2b), plant richness (Fig 2c) or observed interactions (Fig 2d). Finally, rarefaction analysis indicated that sampling of the CC sites was sufficient to capture bird and plant species richness and their interactions (extrapolated curves tend to stability).

**Fig 2:**
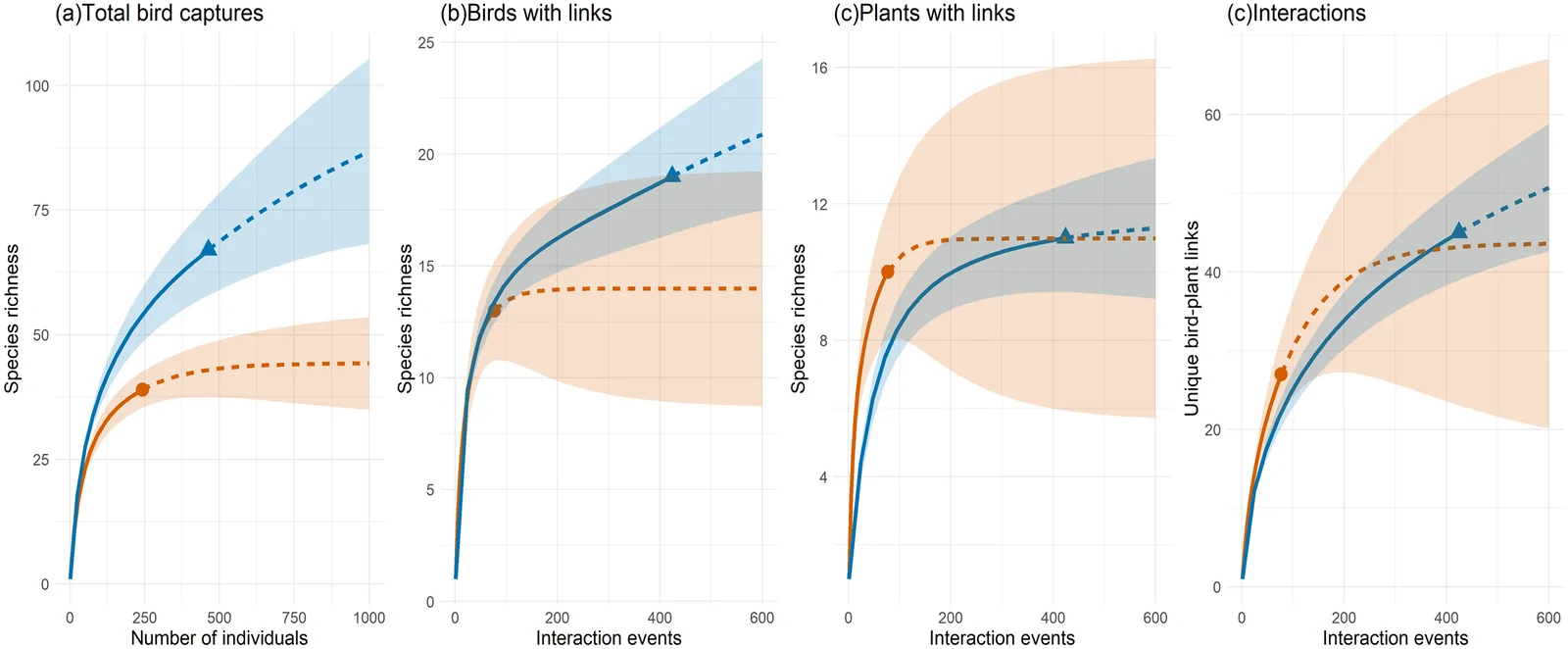
Accumulation curve of species and number of interactions of the total captures and within the frugivorous networks in the secondary araucaria forests at the RPPN Pro-Mata, Rio Grande do Sul, Brazil. Observed values in the clear-cutting (CC) network (blue triangle) and selective logging (SL) network (red circle) are shown. The solid and dashed lines represent the interpolation and extrapolation of richness, respectively. The shaded area surrounding the lines shows the 95% confidence interval.

### Topology of interaction networks

We recorded a total of 501 interaction events involving 23 bird species and 13 plant species. In the CC network, we recorded 424 events, 19 bird species, and 11 plant species. In the SL network, we recorded 77 events, 13 bird species, and 10 plant species.

We found that the *Myrsine lorentziana* plant concentrated the highest number of interactions as a species (Fig 3): 84.7% of interactions in the CC network and 47.7% in the SL network. Similarly, the bird family Turdidae (comprising 5 species) accounted for 51.2% of interactions in the CC network and 63.1% in the SL network (Fig 3).

**Fig 3:**
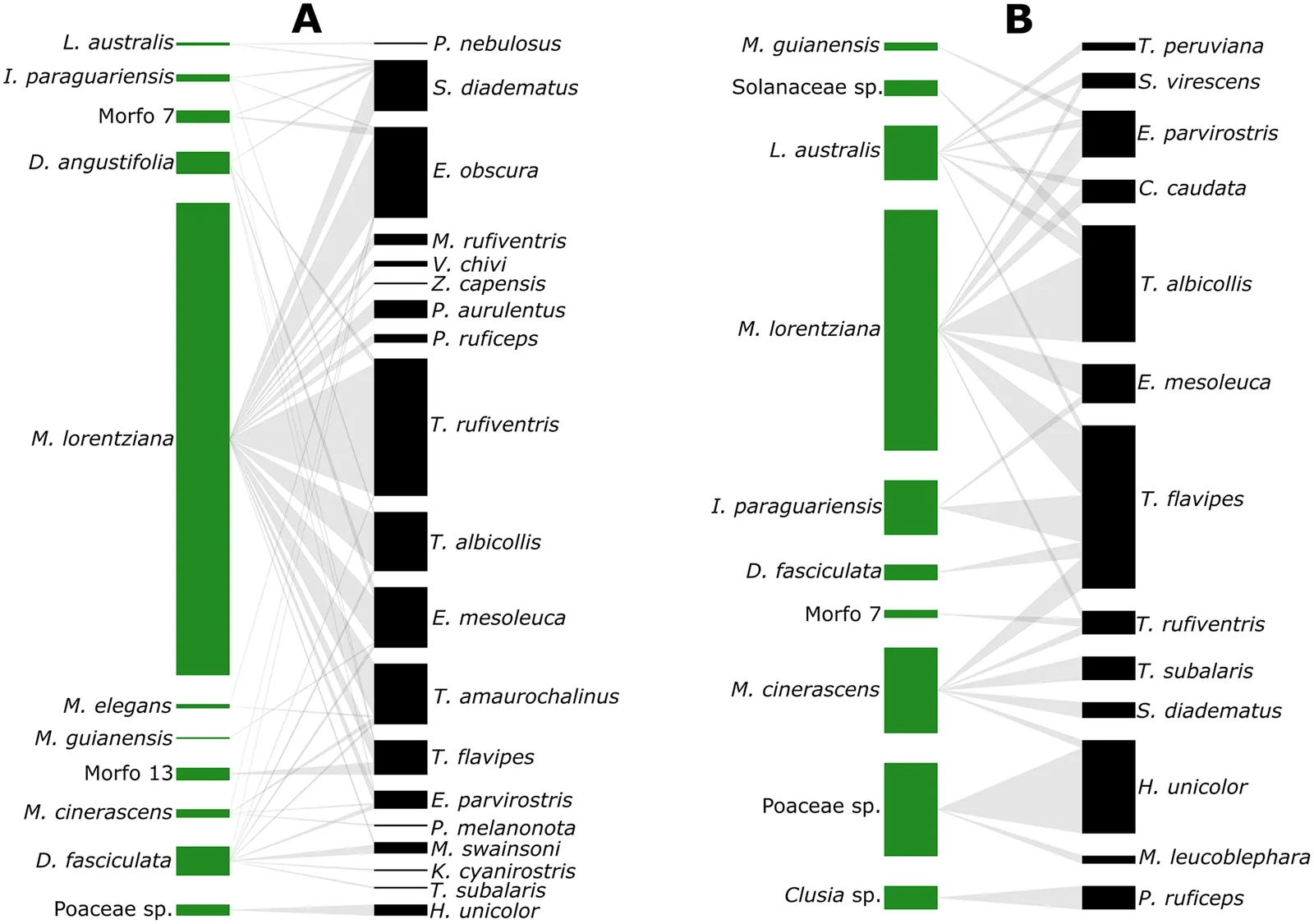
Bipartite graph of plant-frugivorous birds networks in the secondary *Araucaria* Forest at RPPN Pro-Mata, Rio Grande do Sul, Brazil. A. network sampled from forest sites with a history of clear-cut (CC). B. network sampled from forest sites with a history of selective logging (SL). Each axis consists of rectangular nodes representing plant species (green) and bird species (black); the nodes vary in size according to the number of interaction events per species. The size of the links between nodes represents the frequency of recorded events.

At the guild level, in the CC network, omnivores accounted for the largest proportion of interactions (60.4%), followed by invertebrate consumers (21%), frugivores (16.5%), and seed consumers (2.1%). Conversely, in the SL network, both frugivores and invertebrate consumers each accounted for 32.5% of interactions, followed by omnivores (19.5%) and seed consumers (15.6%).

### Network level metrics

The degree of nestedness (NODF) was 46.38 for the CC network and 31.25 for the SL network. According to the null models, both networks did not show nested values (Table 1 and S3-S4 Appendix). Networks from both disturbance histories were highly specialized (*H2’*) and modular (Q), with observed values significantly departing from null models (Table 1). Specialization did not differ significantly between networks (W = 4, p = 0.65), although it was numerically lower in the CC network (*H2’* = 0.40) in comparison with the SL network (*H2’* = 0.56). On the other hand, modularity, according to z-core values, was higher in the CC network (11.55) compared to the SL network (8.75). The CC network was structured into 6 modules comprising 1–9 species (Fig 4) and the SL network was composed of 5 modules containing 1–10 species (Fig 4).

**Table 1:** Values of nestedness (NODF), specialization (*H2’*), modularity (Q), and mean values of the null models generated for networks sampled in of the secondary *Araucaria* Forest at RPPN Pro-Mata, Rio Grande do Sul, Brazil, with history of clear-cutting (CC) and selective logging (SL).

Metrics – networks	Obs.	Zscore	Null model average	Confidence interval (95%)	P-value ^a^
**Nestedness (NODF)**					
**CC**	46.38	−5.72	71.06	70.79-71.33	<0.001
**SL**	31.25	−3.75	58.18	57.73-58.62	<0.001
**Specialization (*H2’*)**					
**CC**	0.40	17.10	0.10	0.10-0.10	<0.001
**SL**	0.56	10.07	0.18	0.18-0.18	<0.001
**Modularity (Q)**					
**CC**	0.17	11.10	0.07	0.07-0.08	<0.001
**SL**	0.50	9.31	0.25	0.25-0.26	<0.001

^a^*p* < 0.05 indicates a significant difference between the observed values and the values obtained from the null models.

**Fig 4:**
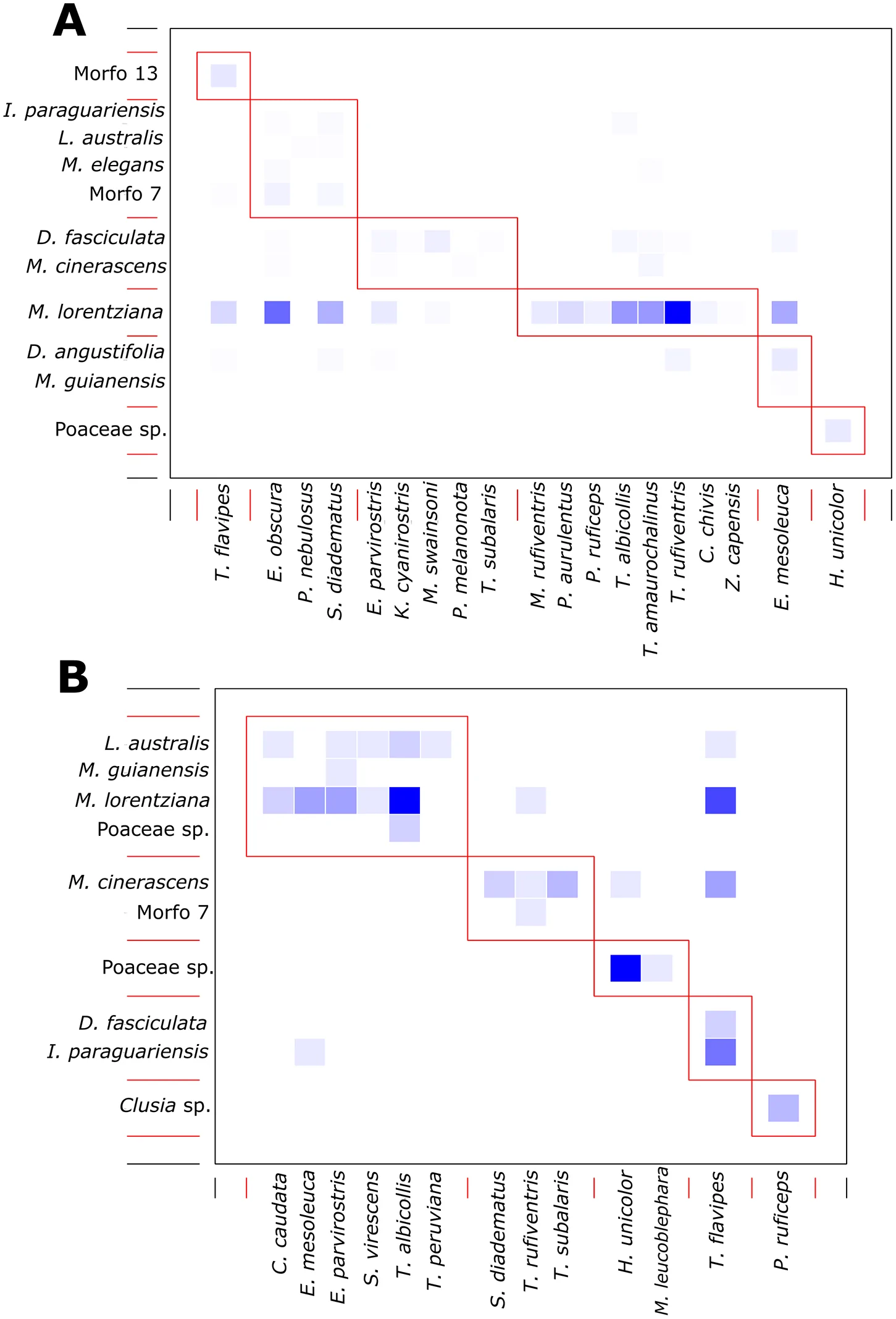
Clustering of plant and bird species into interaction modules (red frames) in the clear-cutting (CC) (A) and selective logging (SL) (B) interaction network of a secondary *Araucaria* Forest at RPPN Pro-Mata, Rio Grande do Sul, Brazil. The squares represent the links between species, with the intensity of the blue color representing the frequency of interaction between species pairs.

The clustering into modules appeared to be related to bird beak width, as we compared this attribute of birds per module and found significant differences in the average beak width per module (Table 2).

**Table 2:** Average, minimum, and maximum values of beak width (mm) of birds per module in each network in of the secondary *Araucaria* Forest at RPPN Pro-Mata, Rio Grande do Sul, Brazil, with history of clear-cutting (CC) and selective logging (SL).

Network	Number of Modules	Average peak width measurement (min.-max.)	Modules with significant difference P < 0.05
CC	1	4.5 mm	–
	2	x̄ = 5.7 mm (3.4–8.3 mm)	4
	3	x̄ = 5.6 mm (5.2–5.8 mm)	4
	4	x̄ = 4.5 mm (4.1–4.8 mm)	2, 3 y 6
	5	x̄ = 5.1 mm (4.2–6.8 mm)	–
	6	x̄ = 5.5 mm (4.1–7.9 mm)	4
SL	1	x̄ = 6.0 (5.3–6.8 mm)	5
	2	4.6 mm	–
	3	x̄ = 6.2 (5.5–6.3 mm)	5
	4	x̄ = 4.9 (3.7–6.3 mm)	–
	5	x̄ = 4.9 (3.7–5.9 mm)	1 y 3

### Species-level metrics

Species-level metrics showed that in the CC network, the plant *Myrsine lorentziana* had the highest normalized degree (0.74), followed by *Elaenia obscura* (0.55) and the plant *Daphnopsis fasciculata* (0.47) (S5 Appendix). Similarly, in the SL network, *M. lorentziana* was the species with the highest normalized degree (0.54), followed by the bird *Turdus flavipes* (0.5) and the plant *Leandra australis* (0.46) (S5 Appendix).

Regarding the degree of specialization (*d’*) we observed that in the CC network the most specialized species were the bird *Haplospiza unicolor* (*d’* = 1), the plant Poaceae sp. (*d’* = 1) and the bird *Picumnus nebulosus* (*d’* = 0.88) (S5 Appendix). On the other hand, in the SL network the species with the greatest specialization were the bird *Pyrrhocoma ruficeps* (*d’* = 1), the plant *Clusia* sp. (*d’* = 1) and the plant Poaceae sp. (*d’* = 0.95) (S5 Appendix).

The degree of bird species in the CC network was positively related to the local abundance of its members (p < 0.05, R^2^ = 0.23, F = 5.01), implying that the number of interactions of a given species was predicted by its abundance in the community in clear-cut successional areas. Conversely, degree was not predicted by abundance in selective logging areas (p > 0.05, R2: 0.10, F:1.17). Additionally, species-level specialization (*d’*) was not predicted by species abundance neither in CC areas (p > 0.05, R2: 0.02, F:0.42) nor in SL areas(p > 0.05, R2: 0.04, F: 0.46).

Species centrality analysis (Gc) indicated a turnover of central and peripheral species from one network to another. In the CC network, we detected 8 central species, where *Myrsine lorentziana* (Gc = 1.78) was the only plant and the species with the highest centrality degree. This species was followed in centrality degree by 7 bird species: *Turdus rufiventris* (Gc = 0.78), *Elaenia obscura* (Gc = 0.78), *T. amaurochalinus* (Gc = 0.22), *E. mesoleuca* (Gc = 0.22), among others (S5 Appendix). Similarly, in the SL network, *M. lorentziana* was the species with the highest centrality (Gc = 1.02), and we identified two other central plant species: Poaceae *sp.* (Gc = 0.19) and *Miconia cinerascens* (Gc = 0.14). Additionally, we found that the bird *T. flavipes* was the second most central species (Gc = 0.14) after *M. lorentziana*, followed by another 3 bird species: *T. albicollis* (Gc = 0.52), *Haplospiza unicolor* (Gc = 0.35), and *E. parvirostris* (Gc = 0.004).

## Discussion

The objective of this research was to compare plant-bird frugivore networks from two secondary forests in recovery from different histories of use, namely clear-cutting (CC) and selective logging (SL). Our results indicated that these disturbance regimes lead to distinct ecological reorganization pathways of mutualistic interactions. Despite these differences, both networks displayed similar low levels of specialization, reflecting a predominance of generalized interactions typical of secondary forests.. The presence of central plant and bird taxa shared between successional stages suggests a degree of functional continuity that may facilitate network persistence during forest recovery. Finally, we detected potentially neutral mechanisms behind the assembly of the clear-cutting network, whereas interactions of the selective logging network were not predicted by abundance, which indicates that niche mechanisms may be more important in this successional environment. Although our results are derived from a specific site in southern Brazil, the observed patterns suggest that disturbance history can shape interaction networks through changes in the relative importance of neutral versus niche mechanisms, which could be extrapolated to similar subtropical forests that encompass a complex mosaic of post-disturbance successional stages.

### Network components by forest type

Our bird records showed differences in species diversity between habitats, with higher records of richness and abundance in the SL forests, surpassing the clear-cut forests by 28 species and 220 individuals. It should be noted that this result is peculiar because it is opposite to the common pattern of finding greater bird diversity in more structurally complex forests [10,64,65]. However, previous works [66,67] also reported greater numbers of species and individuals in less complex forests. Likewise, [68] also found this relationship: greater bird diversity in younger secondary habitats in comparison with primary forests. It is worth mentioning, however, that this inverse relationship could be attributed to the influence of certain plant species [66,67] or to the proximity of primary forest matrices [68] rather than to the physiognomy of the habitat itself [66,67].

The availability of fruits can significantly impact avian community dynamics and ecosystem structure, as demonstrated by [3] in their investigation of the gradient of secondary and primary forests within the Atlantic Forest biome. Their findings revealed that fruit availability played a pivotal role in driving an increase in frugivorous bird populations toward mature forest habitats. Similarly, [19] conducted a study along the succession gradient of Araucaria forests in Santa Catarina state, shedding light on the influence of bamboo cluster fruiting (*Merostachys* aff. *multiramea*) on the richness of granivorous bird species across the landscape. These studies underscore the importance of understanding the intricate relationships between fruit availability, avian foraging behavior, and ecosystem dynamics in forest ecosystems.

In light of our results, we consider that an explanation for the inverse relationship between diversity and habitat may be attributed to the influence of high fruit production during this time of the year in the removal habitat, primarily by the species *Myrsine lorentziana* [9], Although *M. lorentziana* fruits throughout the year, peak fruiting coincides with our sampling period, which typically occurs during the warmer months between summer and early autumn [34], this period overlaps with the breeding season of birds and the arrival of migratory species in southern Brazil [30–33].

### Diversity, composition and representativeness of species

The networks we sampled exhibited different configurations, but in some components, there were no marked differences, such as in bird and plant richness and the number of interactions according to our rarefaction estimates. However, at the species community level, notable differences were observed. One prominent feature was relative importance and centrality of *Myrsine lorentziana* in our frugivory networks. We believe that this representation may be related to its abundance, large fruit output, and dispersal type. *M. lorentziana* shows high density in open areas and early regeneration stages in southern Brazil [9,35,36]. Additionally, it fruits throughout the year with peaks during the warmer months, and its zoochoric dispersal is carried out by birds [34–36].

Another noteworthy finding was that the Turdidae family represented a significant group of birds in the frugivory networks, as the combination of its 5 species concentrated a large proportion of interactions in both networks. his may be linked to their preference for fruit consumption, resulting in their good seed dispersal ability in southern Brazil [69]. Within our networks, we observed that, at the trophic level of birds, two species ranked highest in terms of interaction concentration: *Turdus rufiventris* in the CC network and *T. flavipes* in the SL network. *T. rufiventris* is a thrush with an omnivorous diet [55] found in a wide variety of habitats ranging from forest interiors to open areas, including cities [19,70,71], while *T. flavipes* has a frugivorous diet [55] and is an altitudinal migrant that visits high-elevation regions during spring and summer in southeastern Brazil [33].

Regarding the trophic guilds of birds, the presence of invertebrate consumers in both networks was remarkable. Their presence was not unexpected, as some species include fruits in 20–40% of their diet (e.g., *Elaenia parvirostris*, *Myiarchus swainsoni*, *Vireo chivi*, and *Turdus albicollis* [55]). However, it was noteworthy that species previously categorized as being 100% invertebrate-specialists [55] such as *Picumnus nebulosus*, *Piculus aurulentus*, *Knipolegus cyanirostris*, *Myiothlypis leucoblephara*, and *Pyrrhocoma ruficeps*, had interactions with some plants. Although these species may be participating accidentally in frugivory networks, for example by capturing an insect that was perched on a small fruit and swallowing the fruit in the process, they are nevertheless part of our sampled networks. Another explanation is that those species might have broader diets than previously described, which opens a venue for future studies on their autoecology.

### Network metrics

#### Nestedness.

Regularly, mutualistic networks exhibit nesting as a result of the asymmetric distribution of interactions, where a core of central species concentrates connections among themselves and with peripheral species [27,72]. It is also observed that nestedness can sometimes be lower than values generated at random, indicating random nesting [14,73], as in the case of our research. Some reasons are linked to the study method, which limits the representativeness of the species community [73], on the other hand, it could also be related to the low resilience of species to disturbance in disturbed areas, where specialist species are also scarce [14]. In disturbed successional habitats like the ones reported here, sampling effort could be complemented with additional methodologies aimed at increasing the detection of interactions, such as transects, point counts, and focal observations of fruiting trees, which could ultimately enable the detection of a significantly nested pattern [74]. Alternatively, such systems could, in fact, comprise non-nested mutualistic networks, which structures could shift towards higher nestedness values as successional processes take place with time.

#### Specialization.

The specialization index of networks (*H2’*) indicates the degree of resource overlap exploited by the species community [20,61]. A network with high specialization corresponds to low niche overlap, while low specialization indicates niche convergence [20,61]. In our study, we observed that both networks were more specialized than expected by chance. We observed high resource overlap in the CC network, mainly by birds towards the species *Myrsine lorentziana*, resulting in a lower specialization value compared to the SL network. We found that our networks were very close to the mean specialization values in dispersal networks (*H2’*: 0.29 ± 0.10) previously reported [20]. Mutualistic frugivory networks often show lower specialization compared to mean specialization values in networks involving a higher degree of reciprocal partner specificity, such as pollination networks (*H2’*: 0.55 ± 0.17) or ant-plant networks (*H2’*: 0.68 ± 0.26) [20].

Certain studies of frugivory networks in forest gradients have shown similarities and/or variations from one habitat to another. For instance, in a locality in Kenya, [75] found frugivory networks with similar specialization values between secondary and primary forests. Meanwhile, [7] also noted that specialization was higher in the interior of the forest compared to a more disturbed stage of the forest. At a regional scale, the study by [2] found that the specialization of seed dispersal networks was not altered by the fragmentation gradient, even though other network measures such as species richness and their connections were negatively influenced.

While both neutral and niche processes influence the establishment of mutualistic relationships [22,27,76], our species-level analysis showed that the specialization of birds was not affected by their local abundance. In other words, the most specialized species were not less abundant in the locality, and highly abundant species were not necessarily generalists. In this case, specialization levels would be influenced by factors such as the cohesion of functional traits among species [77], such as morphological congruence between frugivores’ beaks and the size of the fruit they consume [78–82], as well as other niche-related factors such as competitive and facilitative interactions (the latter being less explored in the literature). To enhance the visualization of these relationships, studies of plant-frugivore networks are complemented with functional and phylogenetic ecology approaches, achieving a better understanding of the complexity of relationships [54,83–85] concluding that phenotypic and phenological traits drive generalized and specialized links in community networks [26].

#### Modularity.

The sub-grouping of networks into modules represents a close relationship between partners; the degree of internal clustering of a network can vary and be measured by the metric of modularity [21–23,86]. Modularity can vary greatly among different frugivory networks in the Neotropics [86]. In our research, we detected that both networks were significantly more modular than expected by chance, with the CC network showing a higher absolute modularity score and number of modules in comparison with null models and the SL network. Higher modularity represents an increase in network persistence against gradual extinctions of participating community members [87,88]. In disturbed areas, frugivory networks are also modular and demonstrate the ability to withstand disturbances [89].

Modularity in networks can arise from niche factors such as diet, body size, phylogenetic relationships, or habitat structure of species [90–94]. In our study, we found differences in the mean values of birds’ gape width (Table 2), a variable related to the size of the fruit they ingest [4,79,95], indicating that the compartmentalization of our study networks was likely driven by niche properties. Fruit size or phylogenetic relationships would be variables of interest in future research as they are important in close partner relationships [89,91,96,97].

The central species identified in both networks were part of many detected modules. In the case of the CC network, 4 out of the 6 modules consisted of at least one central species, while the 2 smaller modules (1 plant species and 1 bird species) were purely peripheral. In the case of the SL network, 3 out of the 4 modules had at least one central species, with the smallest module being purely peripheral. Our results reaffirm that mutualistic networks, even if assembled in successional habits with different disturbance histories, are internally organized into modules around central species that maintain the stability of connections [24–26,98].

#### Species centrality.

The core of generalist species in the CC network consisted of only one plant species and six bird species (S5 Appendix), whereas the core of the SL network was composed of four plant species and threebird species (S5 Appendix). It is noteworthy that we detected two species that played central roles as core generalists in both networks, the plant *Myrsine lorentziana* and the bird *Turdus albicollis*. This result suggests that, despite the structural changes in the species community influenced by habitat [10,16,99–102], part of the central species are maintained in space, which agrees with previous findings in a study of ant-plant mutualistic networks on a spatial scale in the Brazilian Amazon [25], or as demonstrated by [103] in their study of bird-plant frugivorous networks on a temporal scale in the Gulf of Mexico.

Dáttilo et al. [25] mention that the persistence of central species in space could be related to species abundance or resource competition among them. In our case, we found that local abundance determined 23% (GLM R^2^ = 0.23) of the interaction degree of bird species in the CC network. However, we did not find this same pattern in the SL network, suggesting that, along with neutral mechanisms, other processes such as niche processes would intervene in shaping the mutualistic network in these particular successional habitats [22,27,76]. Ultimately, our findings point out that the relative contribution of neutral, niche and other processes seems to vary according to the disturbance history.

Migrant species [33,104] also formed part of the core central species in both networks. Among the six central bird species in the CC network, 2 were migrants: *Turdus amaurochalinus* and *Elaenia mesoleuca*. In contrast, in the SL network, among the 3 bird species, 2 were migrants: *T. flavipes and E. parvirostris*. This indicates that our frugivory networks may be affected by seasonality and migration of species to southern Brazil. However, local species of both birds and plants are also an important core of interaction and also provide stability to mutualistic networks [22,24,105]. Although our work does not represent a broad temporal scale, our results suggest that an important event for the structuring of frugivory networks in the secondary araucaria forest would be linked to plant phenology and bird migratory movements.

While our analyses revealed clear structural differences between frugivory networks associated with contrasting disturbance histories, it is important to recognize that the plant-bird interactions documented here represent only a subset of the complex interaction systems within a community. Factors such as climatic conditions or habitat structure were not explicitly quantified and could influence both species presence and interaction patterns. Furthermore, our sampling design captures interactions encompassing seasonal variation in plant phenology and bird movements over a relatively short period. Future studies incorporating longer timescales, additional interaction types, and habitat-level variables will be essential for more accurately interpreting the relative roles of these processes in shaping the organization of mutualistic networks. Additionally, including phylogenetic analyses would allow for progress toward an evolutionary and functional interpretation of the observed patterns.

## Conclusions

Our research can help understand the role of birds on the regeneration and conservation of Araucaria forests in southern Brazil. We provide further evidence that birds are connecting a variety of habitats in a diverse successional mosaic via use of plant resources, with direct implications as modulators of forest successional dynamics and potential promoters of forest expansion over natural non-forest ecosystems (i.e., grasslands) present in the region. While mutualistic plant-bird frugivory networks from different disturbance histories and successional trajectories showed structural differences in network metrics, they also showed important similarities, such as shared core species in both trophic levels. Specifically, we emphasize the relevance of *Myrsine lorentziana*, the most important plant in networks with strikingly distinct successional histories and properties. This species is a known pioneer that starts forest nuclei in open areas [106,107], and seems to play a key role in connecting different successional forest stages through frugivory interactions. Taken together, these findings highlight the potential relevance of early successional forest areas as forage sources for frugivorous birds, for the maintenance of more advanced successional areas and, ultimately, the conservation of these ecosystems. Forest mosaics with secondary areas under different successional trajectories are arguably the most common landscape configuration in tropical and subtropical ecosystems [108–111]. Focusing on conserving these mosaics, as opposed to relying on ‘pristine’ advanced successional areas alone, seem to be the optimal strategy for maintaining and potentially maximizing species diversity [112] and a fundamental mutualistic system [1,78,113–115] in these highly fragmented landscapes.

## Supporting information

S1 Appendix List of captured species and number of interaction events in of the secondary *Araucaria* Forest at RPPN Pro-Mata, Rio Grande do Sul, Brazil, with history of clear-cutting (CC) and selective logging (SL). (DOCX)

S2 Appendix List of plant species consumed by birds in of the secondary *Araucaria* Forest at RPPN Pro-Mata, Rio Grande do Sul, Brazil, with history of clear-cutting (CC) and selective logging (SL). (DOCX)

S3 Appendix Results of null model algorithms (“swap.web,” “vaznull,” “shuffle.web,” and “mgen”) to evaluate the presence of nesting with the índices nestedness, NODF, weighted nestedness and weighted NODF. (DOCX)

S4 Appendix Graphs showing a comparison between the observed value of the NODF index (Nestedness metric based on Overlap and Decreasing Fill) and the distribution of expected values under a null model, generated using method 1, 2, 3, 4 and 5 of the bipartite package in R. X-axis: represents the values of the NODF index, which measures the degree of network nestedness; Y-axis: represents the probability density of the NODF values simulated under the null model; Black curve: shows the distribution of NODF values randomly generated by the null model; and Blue vertical line: indicates the observed NODF value for the empirical network.
(DOCX)

S5 Appendix Values of the normalize degree (Nd), specialization (d’) and degree centrality (Gc) indices in the frugivore networks in of the secondary *Araucaria* Forest at RPPN Pro-Mata, Rio Grande do Sul, Brazil, with history of clear-cutting (CC) and selective logging (SL). (DOCX)
