# Supplementary material for: Bird-plant frugivory networks in two secondary Araucaria forests in southern Brazil
Source: PLoS One. 2026 Sep 30;21(9):e0357566. doi: 10.1371/journal.pone.0357566 (PMC13626356; doi:10.1371/journal.pone.0357566)
Supplement: Supplementary file 1 [file pone.0357566.s001.docx]

**S1 Appendix. List of captured species and number of interaction events in of the secondary *Araucaria* Forest at RPPN Pro-Mata, Rio Grande do Sul, Brazil, with history of clear-cutting (CC) and selective logging (SL).**

| **Scientific name** | **Diet (Wilman et al., 2014)** | **Individuals/interaction events** | |
| --- | --- | --- | --- |
|  |  | **CC network** | **SL network** |
| **Columbiformes (2)** |  |  |  |
| **Columbidae (2)** |  |  |  |
| *Leptotila verreauxi* | Seeds | 2/0 | 1/0 |
| *Leptotila rufaxilla* | Seeds | 3/0 | 1/0 |
| **Piciformes (3)** |  |  |  |
| **Picidae (3)** |  |  |  |
| *Picumnus nebulosus* | Invertebrates | 6/1 | 0 |
| *Veniliornis spilogaster* | Invertebrates | 2/0 | 0 |
| *Piculus aurulentus* | Invertebrates | 3/13 | 0 |
| **Passeriformes (69)** |  |  |  |
| **Thamnophilidae (4)** |  |  |  |
| *Thamnophilus ruficapillus* | Invertebrates | 1/0 | 0 |
| *Thamnophilus caerulescens* | Invertebrates | 5/0 | 0 |
| *Mackenziaena leachii* | Invertebrates | 1/0 | 0 |
| *Pyriglena leucoptera* | Invertebrates | 1/0 | 0 |
| **Conopophagidae (1)** |  |  |  |
| *Conopophaga lineata* | Invertebrates | 1/0 | 6/0 |
| **Grallaridae (1)** |  |  |  |
| *Hylopezus nathereri* | Invertebrates | 1/0 | 0 |
| **Scleruridae (1)** |  |  |  |
| *Sclerurus scansor* | Invertebrates | 0 | 3/0 |
| **Dendrocolaptidae (6)** |  |  |  |
| *Sittasomus griseicapillus* | Invertebrates | 1/0 | 4/0 |
| *Xiphorhynchus fuscus* | Invertebrates | 0 | 2/0 |
| *Campylorhamphus falcularius* | Invertebrates | 0 | 1/0 |
| *Lepidocolaptes falcinellus* | Invertebrates | 1/0 | 2/0 |
| *Dendrocolaptes platyrostris* | Invertebrates | 1/0 | 2/0 |
| *Xiphocolaptes albicollis* | Invertebrates | 0 | 2/0 |
| **Furnariidae (8)** |  |  |  |
| *Philydor rufum* | Invertebrates | 1/0 | 1/0 |
| *Heliobletus contaminatus* | Invertebrates | 3/0 | 6/0 |
| *Syndactyla rufosuperciliata* | Invertebrates | 7/0 | 12/0 |
| *Leptasthenura striolata* | Invertebrates | 4/0 | 0 |
| *Cranioleuca obsoleta* | Invertebrates | 1/0 | 0 |
| *Synallaxis ruficapilla* | Invertebrates | 0 | 2/0 |
| *Synallaxis cinerascens* | Invertebrates | 3/0 | 8/0 |
| *Synallaxis spixi* | Invertebrates | 4/0 | 0 |
| **Pipridae (1)** |  |  |  |
| *Chiroxiphia caudata* | Omnivore | 1/0 | 3/3 |
| **Tityridae (1)** |  |  |  |
| *Schiffornis virescens* | Omnivore | 0 | 5/2 |
| **Cotingidae (1)** |  |  |  |
| *Carpornis cucullata* | Frugívoro | 6/0 | 0 |
| **Platyrinchidae (1)** |  |  |  |
| *Platyrinchus mystaceus* | Invertebrates | 3/0 | 17/0 |
| **Rhynchocyclidae (3)** |  |  |  |
| *Mionectes rufiventris* | Omnivore | 10/8 | 0 |
| *Phylloscartes ventralis* | Invertebrates | 17/0 | 0 |
| *Hemitriccus obsoletus* | Invertebrates | 1/0 | 9/0 |
| **Tyrannidae (15)** |  |  |  |
| *Tyranniscus burmeisteri* | Omnivore | 1/0 | 0 |
| *Camptostoma obsoletum* | Invertebrates | 1/0 | 0 |
| *Elaenia parvirostris* | Invertebrates | 15/13 | 5/6 |
| *Elaenia mesoleuca* | Omnivore | 26/44 | 6/5 |
| *Elaenia obscura* | Omnivore | 19/66 | 0 |
| *Phyllomyias virescens* | Invertebrates | 7/0 | 1/0 |
| *Serpophaga subcristata* | Invertebrates | 3/0 | 0 |
| *Attila phoenicurus* | Invertebrates | 1/0 | 0 |
| *Attila rufus* | Invertebrates | 1/0 | 0 |
| *Myiarchus swainsoni* | Invertebrates | 19/8 | 3/0 |
| *Myiophobus fasciatus* | Invertebrates | 14/0 | 0 |
| *Lathrotriccus euleri* | Invertebrates | 5/0 | 7/0 |
| *Knipolegus cyanirostris* | Invertebrates | 15/1 | 9/0 |
| *Knipolegus nigerrimus* | Invertebrates | 1/0 | 0 |
| *Muscipipra vetula* | Invertebrates | 3/0 | 0 |
| **Vireonidae (3)** |  |  |  |
| *Cyclarhis gujanensis* | Omnivore | 2/0 | 3/0 |
| *Hylophilus poicilotis* | Invertebrates | 3/0 | 0 |
| *Vireo chivi* | Invertebrates | 23/4 | 0 |
| **Turdidae (5)** |  |  |  |
| *Turdus flavipes* | Frugívoro | 13/25 | 27/21 |
| *Turdus rufiventris* | Omnivore | 38/100 | 15/3 |
| *Turdus amaurochalinus* | Frugívoro | 29/44 | 0 |
| *Turdus subalaris* | Frugívoro | 3/1 | 2/3 |
| *Turdus albicollis* | Invertebrates | 8/43 | 6/15 |
| **Passerellidae (1)** |  |  |  |
| *Zonotrichia capensis* | Seeds | 10/1 | 0 |
| **Parulidae (4)** |  |  |  |
| *Setophaga pitiayumi* | Invertebrates | 1/0 | 0 |
| *Geothlypis aequinoctialis* | Invertebrates | 11/0 | 0 |
| *Basileuterus culicivorus* | Invertebrates | 3/0 | 5/0 |
| *Myiothlypis leucoblephara* | Invertebrates | 13/1 | 36/0 |
| **Thraupidae (12)** |  |  |  |
| *Pipraeidea melanonota* | Omnivore | 1/1 | 0 |
| *Stephanophorus diadematus* | Omnivore | 23/37 | 5/2 |
| *Tangara preciosa* | Frugívoro | 1/0 | 1/0 |
| *Haplospiza unicolor* | Seeds | 26/8 | 12/12 |
| *Hemithraupis guira* | Invertebrates | 1/0 | 0 |
| *Tachyphonus coronatus* | Invertebrates | 2/0 | 0 |
| *Dacnis cayana* | Omnivore | 1/0 | 0 |
| *Saltator similis* | Invertebrates | 5/0 | 1/0 |
| *Saltator maxillosus* | Invertebrates | 2/0 | 0 |
| *Poospiza thoracica* | Omnivore | 3/0 | 0 |
| *Microspingus cabanisi* | Omnivore | 15/0 | 4/0 |
| *Pyrrhocoma ruficeps* | Invertebrates | 5/6 | 7/3 |
| **Cardinalidae (1)** |  |  |  |
| *Amaurospiza moesta* | Seeds | 0 | 1/0 |
