# Supplementary material for: Bird-plant frugivory networks in two secondary Araucaria forests in southern Brazil
Source: PLoS One. 2026 Sep 30;21(9):e0357566. doi: 10.1371/journal.pone.0357566 (PMC13626356; doi:10.1371/journal.pone.0357566)
Supplement: Supplementary file 2 [file pone.0357566.s002.docx]

**S2 Appendix. List of plant species consumed by birds in of the secondary *Araucaria* Forest at RPPN Pro-Mata, Rio Grande do Sul, Brazil, with history of clear-cutting (CC) and selective logging (SL).**

| Scientific name | No. Seeds/interaction events | |
| --- | --- | --- |
|  | CC network | SL network |
| Aquifoliales (1) |  |  |
| Aquifoliaceae (1) |  |  |
| *Ilex paraguariensis* | 13/5 | 20/7 |
| Canellales (1) |  |  |
| Winteraceae (1) |  |  |
| *Drimys angustifolia* | 49/16 | 0 |
| Ericales (1) |  |  |
| Primulaceae (1) |  |  |
| *Myrsine lorentziana* | 344/344 | 31/31 |
| Orden Indeterminado (2) |  |  |
| Familia Indeterminada (2) |  |  |
| Morfo 13 | 9/9 | 0 |
| Morfo 7 | 38/9 | 2/1 |
| Laurales (1) |  |  |
| Monimiaceae (1) |  |  |
| *Mollinedia elegans* | 3/3 | 0 |
| Malpighiales (1) |  |  |
| Clusiaceae (1) |  |  |
| *Clusia sp*. | 0 | 11/3 |
| Malvales (1) |  |  |
| Thymelaeaceae (1) |  |  |
| *Daphnopsis fasciculata* | 21/21 | 2/2 |
| Myrtales (3) |  |  |
| Melastomataceae (2) |  |  |
| *Leandra australis* | 45/2 | 57/7 |
| *Miconia cinerascens* | 24/6 | 44/11 |
| Myrtaceae (1) |  |  |
| *Myrcia guianensis* | 1/1 | 1/1 |
| Poales (1) |  |  |
| Poaceae (1) |  |  |
| Poaceae sp. | 8/8 | 12/12 |
| Solanales (1) |  |  |
| Solanaceae (1) |  |  |
| Solanaceae sp. | 0 | 5/2 |
