# Supplementary material for: Bird-plant frugivory networks in two secondary Araucaria forests in southern Brazil
Source: PLoS One. 2026 Sep 30;21(9):e0357566. doi: 10.1371/journal.pone.0357566 (PMC13626356; doi:10.1371/journal.pone.0357566)
Supplement: Supplementary file 3 [file pone.0357566.s003.docx]

**S3 Appendix. Results of null model algorithms ("swap.web", "vaznull", "shuffle.web", and "mgen") to evaluate the presence of nesting with the índices nestedness, NODF, weighted nestedness and weighted NODF**.

**Nestedness (CC)**

| Method | Obs | Z-score | Mean.null | Min.null | Max.null | SD.null | UpperCI | LowerCI | P-value |
| --- | --- | --- | --- | --- | --- | --- | --- | --- | --- |
| 1 | 18.35 | 2.15 | 12.32 | 6.45 | 21.36 | 2.81 | 12.49 | 12.14 | 0.022 |
| 2 | 18.46 | 0.99 | 15.56 | 9.36 | 27.51 | 2.93 | 15.74 | 15.38 | 0.176 |
| 3 | 19.47 | 2.09 | 13.51 | 8.50 | 24.71 | 2.85 | 13.69 | 13.34 | 0.041 |
| 4 | 19.71 | -4.08 | 47.87 | 28.09 | 73.26 | 6.90 | 48.30 | 47.45 | 0 |
| 5 | 19.47 | 1.75 | 13.51 | 5.76 | 31.01 | 3.41 | 13.72 | 13.29 | 0.047 |

**NODF (CC)**

| Method | Obs | Z-score | Mean.null | Min.null | Max.null | SD.null | UpperCI | LowerCI | P-value |
| --- | --- | --- | --- | --- | --- | --- | --- | --- | --- |
| 1 | 46.38 | -5.85 | 71.05 | 56.30 | 82.13 | 4.22 | 71.31 | 70.79 | 0 |
| 2 | 46.38 | -1.86 | 54.16 | 39.70 | 65.14 | 4.18 | 54.42 | 53.90 | 0.035 |
| 3 | 46.38 | -2.62 | 57.57 | 41.35 | 68.27 | 4.26 | 57.83 | 57.30 | 0.012 |
| 4 | 46.38 | 10.54 | 20.13 | 11.67 | 30.76 | 2.49 | 20.28 | 19.97 | 0 |
| 5 | 46.38 | -6.11 | 72.12 | 56.96 | 83.54 | 4.21 | 72.38 | 71.85 | 0 |

**Weighted Nestedness (CC)**

| Method | Obs | Z-score | Mean.null | Min.null | Max.null | SD.null | UpperCI | LowerCI | P-value |
| --- | --- | --- | --- | --- | --- | --- | --- | --- | --- |
| 1 | 0.5212 | -6.07 | 0.8660 | 0.6404 | 0.9770 | 0.0568 | 0.8695 | 0.8624 | 0 |
| 2 | 0.5232 | -3.51 | 0.7716 | 0.4812 | 0.9314 | 0.0707 | 0.7760 | 0.7672 | 0.003 |
| 3 | 0.5278 | -5.36 | 0.8370 | 0.5877 | 0.9634 | 0.0577 | 0.8406 | 0.8334 | 0 |
| 4 | 0.5275 | 5.45 | -0.0053 | -0.3330 | 0.2952 | 0.0978 | 0.0008 | -0.0114 | 0 |
| 5 | 0.5231 | -6.31 | 0.8579 | 0.6047 | 0.9730 | 0.0531 | 0.8611 | 0.8546 | 0 |

**Weighted NODF (CC)**

| Method | Obs | Z-score | Mean.null | Min.null | Max.null | SD.null | UpperCI | LowerCI | P-value |
| --- | --- | --- | --- | --- | --- | --- | --- | --- | --- |
| 1 | 32.09 | -4.88 | 53.96 | 33.38 | 64.55 | 4.48 | 54.24 | 53.68 | 0 |
| 2 | 32.09 | -2.61 | 43.35 | 25.24 | 54.07 | 4.32 | 43.61 | 43.08 | 0.006 |
| 3 | 32.09 | -3.44 | 47.86 | 31.75 | 62.28 | 4.59 | 48.14 | 47.57 | 0.001 |
| 4 | 32.09 | 11.95 | 8.46 | 3.09 | 15.48 | 1.98 | 8.58 | 8.34 | 0 |
| 5 | 32.09 | -4.23 | 52.37 | 34.14 | 67.42 | 4.79 | 52.66 | 52.07 | 0 |

**Nestedness (SL)**

| Method | Obs | Z-score | Mean.null | Min.null | Max.null | SD.null | UpperCI | LowerCI | P-value |
| --- | --- | --- | --- | --- | --- | --- | --- | --- | --- |
| 1 | 28.17 | 2.01 | 17.52 | 6.72 | 41.76 | 5.29 | 17.85 | 17.19 | 0.038 |
| 2 | 28.17 | 1.79 | 20.22 | 12.53 | 40.01 | 4.44 | 20.50 | 19.95 | 0.052 |
| 3 | 28.46 | 1.62 | 20.60 | 12.12 | 39.61 | 4.86 | 20.90 | 20.30 | 0.066 |
| 4 | 27.71 | -2.27 | 45.57 | 24.15 | 70.88 | 7.85 | 46.06 | 45.08 | 0.003 |
| 5 | 28.17 | 1.02 | 21.52 | 7.70 | 46.28 | 6.55 | 21.93 | 21.12 | 0.15 |

**NODF (SL)**

| Method | Obs | Z-score | Mean.null | Min.null | Max.null | SD.null | UpperCI | LowerCI | P-value |
| --- | --- | --- | --- | --- | --- | --- | --- | --- | --- |
| 1 | 31.25 | -3.66 | 58.16 | 37.98 | 82.43 | 7.35 | 58.61 | 57.70 | 0 |
| 2 | 31.25 | -0.53 | 33.70 | 19.11 | 50.00 | 4.67 | 33.99 | 33.41 | 0.307 |
| 3 | 31.25 | -0.47 | 33.85 | 16.40 | 51.96 | 5.61 | 34.20 | 33.51 | 0.321 |
| 4 | 31.25 | 4.34 | 17.40 | 7.18 | 29.61 | 3.19 | 17.60 | 17.20 | 0 |
| 5 | 31.25 | -3.52 | 56.33 | 35.49 | 79.13 | 7.12 | 56.78 | 55.89 | 0 |

**Weighted Nestedness (SL)**

| Method | Obs | Z-score | Mean.null | Min.null | Max.null | SD.null | UpperCI | LowerCI | P-value |
| --- | --- | --- | --- | --- | --- | --- | --- | --- | --- |
| 1 | 0.356 | -2.19 | 0.604 | 0.198 | 0.896 | 0.113 | 0.611 | 0.597 | 0.029 |
| 2 | 0.353 | -1.49 | 0.509 | 0.046 | 0.795 | 0.105 | 0.516 | 0.503 | 0.063 |
| 3 | 0.354 | -1.46 | 0.521 | 0.131 | 0.878 | 0.115 | 0.529 | 0.514 | 0.076 |
| 4 | 0.356 | 3.65 | -0.003 | -0.274 | 0.318 | 0.099 | 0.003 | -0.009 | 0 |
| 5 | 0.360 | -1.50 | 0.538 | 0.122 | 0.894 | 0.119 | 0.546 | 0.531 | 0.083 |

**Weighted NODF (SL)**

| Method | Obs | Z-score | Mean.null | Min.null | Max.null | SD.null | UpperCI | LowerCI | P-value |
| --- | --- | --- | --- | --- | --- | --- | --- | --- | --- |
| 1 | 14.53 | -3.16 | 30.67 | 12.38 | 50.20 | 5.10 | 30.98 | 30.35 | 0.001 |
| 2 | 14.53 | -1.50 | 20.45 | 7.72 | 33.67 | 3.94 | 20.70 | 20.21 | 0.056 |
| 3 | 14.53 | -1.74 | 23.78 | 10.57 | 41.80 | 5.32 | 24.11 | 23.45 | 0.036 |
| 4 | 14.53 | 3.46 | 6.46 | 0.81 | 15.04 | 2.33 | 6.60 | 6.32 | 0.001 |
| 5 | 14.53 | -2.64 | 27.53 | 11.79 | 46.37 | 4.92 | 27.84 | 27.23 | 0.002 |
