# Supplementary material for: Bird-plant frugivory networks in two secondary Araucaria forests in southern Brazil
Source: PLoS One. 2026 Sep 30;21(9):e0357566. doi: 10.1371/journal.pone.0357566 (PMC13626356; doi:10.1371/journal.pone.0357566)
Supplement: Supplementary file 4 [file pone.0357566.s004.docx]

**S4 Appendix. Graphs showing a comparison between the observed value of the NODF index (Nestedness metric based on Overlap and Decreasing Fill) and the distribution of expected values under a null model, generated using method 1, 2, 3, 4 and 5 of the bipartite package in R.** X-axis: represents the values of the NODF index, which measures the degree of network nestedness; Y-axis: represents the probability density of the NODF values simulated under the null model; Black curve: shows the distribution of NODF values randomly generated by the null model; and Blue vertical line: indicates the observed NODF value for the empirical network.
