# Supplementary material for: Bird-plant frugivory networks in two secondary Araucaria forests in southern Brazil
Source: PLoS One. 2026 Sep 30;21(9):e0357566. doi: 10.1371/journal.pone.0357566 (PMC13626356; doi:10.1371/journal.pone.0357566)
Supplement: Supplementary file 5 [file pone.0357566.s005.docx]

**S5 Appendix. Values of the normalize degree (Nd), specialization (d’) and degree centrality (Gc) indices in the frugivore networks in of the secondary *Araucaria* Forest at RPPN Pro-Mata, Rio Grande do Sul, Brazil, with history of clear-cutting (CC) and selective logging (SL).**

| No | Scientific name | Group | Nd | d’ | Gc | No | Scientific name | Group | Nd | d’ | Gc |
| --- | --- | --- | --- | --- | --- | --- | --- | --- | --- | --- | --- |
| CC Network | | | | | | SL Network | | | | | |
| 1 | *Myrsine lorentziana* | Plant | 0.74 | 0.21 | 1.78 | 1 | *Myrsine lorentziana* | Plant | 0.54 | 0.41 | 1.02 |
| 2 | *Turdus rufiventris* | Bird | 0.27 | 0.09 | 0.78 | 2 | *Turdus flavipes* | Bird | 0.50 | 0.33 | 0.86 |
| 3 | *Elaenia obscura* | Bird | 0.55 | 0.08 | 0.44 | 3 | *Turdus albicollis* | Bird | 0.30 | 0.40 | 0.52 |
| 4 | *Elaenia mesoleuca* | Bird | 0.36 | 0.11 | 0.22 | 4 | *Haplospiza unicolor* | Bird | 0.20 | 0.84 | 0.35 |
| 5 | *Turdus amaurochalinus* | Bird | 0.36 | 0.07 | 0.22 | 5 | Poaceae *sp.* | Plant | 0.15 | 0.95 | 0.19 |
| 6 | *Turdus subalaris* | Bird | 0.09 | 0.48 | 0.21 | 6 | *Miconia cinerascens* | Plant | 0.38 | 0.46 | 0.14 |
| 7 | *Turdus albicollis* | Bird | 0.27 | 0.06 | 0.21 | 7 | *Elaenia parvirostris* | Bird | 0.30 | 0.29 | 0.004 |
| 8 | *Stephanophorus diadematus* | Bird | 0.45 | 0.08 | 0.15 | 8 | *Ilex paraguariensis* | Plant | 0.15 | 0.38 | -0.03 |
| 9 | *Turdus flavipes* | Bird | 0.36 | 0.28 | 0.03 | 9 | *Leandra australis* | Plant | 0.46 | 0.27 | -0.03 |
| 10 | *Elaenia parvirostris* | Bird | 0.36 | 0.07 | -0.09 | 10 | *Elaenia mesoleuca* | Bird | 0.20 | 0.18 | -0.05 |
| 11 | *Piculus aurulentus* | Bird | 0.09 | 0.03 | -0.09 | 11 | *Pyrrhocoma ruficeps* | Bird | 0.10 | 1.00 | -0.17 |
| 12 | *Daphnopsis fasciculata* | Plant | 0.47 | 0.39 | -0.10 | 12 | *Turdus subalaris* | Bird | 0.10 | 0.53 | -0.17 |
| 13 | *Drimys angustifolia* | Plant | 0.26 | 0.24 | -0.13 | 13 | *Turdus rufiventris* | Bird | 0.30 | 0.30 | -0.17 |
| 14 | *Haplospiza unicolor* | Bird | 0.09 | 1.00 | -0.14 | 14 | *Chiroxiphia caudata* | Bird | 0.20 | 0.11 | -0.17 |
| 15 | *Myiarchus swainsoni* | Bird | 0.18 | 0.41 | -0.14 | 15 | *Clusia.sp.* | Plant | 0.08 | 1.00 | -0.21 |
| 16 | *Mionectes rufiventris* | Bird | 0.09 | 0.01 | -0.14 | 16 | *Stephanophorus diadematus* | Bird | 0.10 | 0.42 | -0.22 |
| 17 | *Pyrrhocoma ruficeps* | Bird | 0.09 | 0.00 | -0.16 | 17 | *Schiffornis virescens* | Bird | 0.20 | 0.09 | -0.22 |
| 18 | Morfo 13 | Plant | 0.05 | 0.72 | -0.17 | 18 | Solanaceae *sp.* | Plant | 0.08 | 0.30 | -0.25 |
| 19 | Morfo 7 | Plant | 0.16 | 0.28 | -0.17 | 19 | *Daphnopsis fasciculata* | Plant | 0.08 | 0.18 | -0.25 |
| 20 | Poaceae *sp.* | Plant | 0.05 | 1.00 | -0.18 | 20 | *Tangara peruviana* | Bird | 0.10 | 0.43 | -0.28 |
| 21 | *Vireo chivi* | Bird | 0.09 | 0.00 | -0.18 | 21 | *Myiothlypis leucoblephara* | Bird | 0.10 | 0.28 | -0.28 |
| 22 | *Miconia cinerascens* | Plant | 0.21 | 0.38 | -0.19 | 22 | Morfo7 | Plant | 0.08 | 0.64 | -0.29 |
| 23 | *Ilex paraguariensis* | Plant | 0.16 | 0.20 | -0.20 | 23 | *Myrcia guianensis* | Plant | 0.08 | 0.41 | -0.29 |
| 24 | *Mollinedia elegans* | Plant | 0.11 | 0.14 | -0.21 |  |  |  |  |  |  |
| 25 | *Leandra australis* | Plant | 0.11 | 0.59 | -0.21 |  |  |  |  |  |  |
| 26 | *Picumnus nebulosus* | Bird | 0.09 | 0.88 | -0.21 |  |  |  |  |  |  |
| 27 | *Pipraeidea melanonota* | Bird | 0.09 | 0.69 | -0.21 |  |  |  |  |  |  |
| 28 | *Knipolegus cyanirostris* | Bird | 0.09 | 0.48 | -0.21 |  |  |  |  |  |  |
| 29 | *Zonotrichia capensis* | Bird | 0.09 | 0.00 | -0.21 |  |  |  |  |  |  |
| 30 | *Myrcia guianensis* | Plant | 0.05 | 0.18 | -0.22 |  |  |  |  |  |  |
